# The Causal Efficacy of Consciousness

**DOI:** 10.3390/e22080823

**Published:** 2020-07-28

**Authors:** Matthew Owen

**Affiliations:** 1 Yakima Valley College, Yakima, WA 98902, USA; mowen@yvcc.edu; 2 Center for Consciousness Science, University of Michigan Medical School, Ann Arbor, MI 48109, USA

**Keywords:** mental causation, integrated information theory, consciousness, physicalism, monism, physical causal closure, causal exclusion, causal pairing, psychophysical laws

## Abstract

Mental causation is vitally important to the integrated information theory (IIT), which says consciousness exists since it is causally efficacious. While it might not be directly apparent, metaphysical commitments have consequential entailments concerning the causal efficacy of consciousness. Commitments regarding the ontology of consciousness and the nature of causation determine which problem(s) a view of consciousness faces with respect to mental causation. Analysis of mental causation in contemporary philosophy of mind has brought several problems to the fore: the alleged lack of psychophysical laws, the causal exclusion problem, and the causal pairing problem. This article surveys the threat each problem poses to IIT based on the different metaphysical commitments IIT theorists might make. Distinctions are made between what I call reductive IIT, non-reductive IIT, and non-physicalist IIT, each of which make differing metaphysical commitments regarding the ontology of consciousness and nature of causation. Subsequently, each problem pertaining to mental causation is presented and its threat, or lack thereof, to each version of IIT is considered. While the lack of psychophysical laws appears unthreatening for all versions, reductive IIT and non-reductive IIT are seriously threatened by the exclusion problem, and it is difficult to see how they could overcome it while maintaining a commitment to the causal closure principle. Yet, non-physicalist IIT denies the principle but is therefore threatened by the pairing problem, to which I have elsewhere provided a response that is briefly outlined here. This problem also threatens non-reductive IIT, but unlike non-physicalist IIT it lacks an evident response. The ultimate aim of this survey is to provide a roadmap for IIT theorists through the maze of mental causation, by clarifying which commitments lead to which problems, and how they might or might not be overcome. Such a survey can aid IIT theorists as they further develop and hone the metaphysical commitments of IIT.

## 1. Introduction

The causal efficacy of consciousness is vital to the integrated information theory (IIT) of consciousness. IIT opposes eliminativist and illusionist views that deny the ontological existence of consciousness, claiming to the contrary that consciousness is a real feature of the natural world [1], p. 5 (see also [2], p. 3). However, according to IIT, whatever knowingly exists must have causal power that produces physical effects [1], p. 7. As Grasso points out, IIT embraces the Eleatic principle, according to which, what exists has causal power [3], p. 52 (see also [2], p. 81; [4], fn 7). More precisely, IIT’s existence criterion says “…existence requires having maximally irreducible cause-effect power…” [4], sect. Extensions (see also [5]). Given this criterion for existence, if consciousness is devoid of causal power, then it does not exist. Moreover, such a consequence contradicts IIT’s first axiom: “Consciousness *exists*: each experience is *actual*—indeed, that my experience here and now exists (it is real) is the only fact I can be sure of immediately and absolutely” [4], sect. Axioms. Consequently, IIT has a vested interest in mental causation.

It is often assumed that there is only one problem regarding mental causation and that it is a problem for Descartes’ dualism. However, debates in contemporary philosophy of mind concerning mental causation have revealed that physicalist principles about the mental and causation also cause trouble (see [6], p. 9). As it turns out, there are multiple problems of mental causation, each one being generated by different philosophical commitments (see [7], p. 29). Additionally, all views of consciousness implicitly or explicitly entail philosophical commitments that determine which problems they face. This article focuses specifically on which problems IIT faces depending on its potential philosophical commitments. 

To IIT’s credit, its epistemic methodology of starting with axioms about the nature of consciousness makes the theory’s pre-empirical philosophical commitments more explicit and therefore easier to analyze (see [8], pp. 12–13). Yet, the theory is still developing metaphysically, and there are different philosophical commitments that it might make. As discussed in the following section, one aspect of IIT needing further clarity is the ontological identity of consciousness. The subsequent section presents three different possible versions of IIT—reductive IIT, non-reductive IIT, and non-physicalist IIT—which understand the ontology of conscious in varying ways and make different philosophical commitments. After the three positions are presented, the three problems of mental causation are explicated, beginning with the problem of a lack of psychophysical laws followed by the causal exclusion problem before the familiar causal pairing problem is briefly outlined. As each problem is considered, I discuss the threat that it poses to the various versions of IIT and how they can or cannot overcome the problem. 

Elsewhere I have argued for a way that IIT might overcome the causal pairing problem [9]. Therefore, this work gives less attention to the pairing problem, only briefly outlining it and the response I have previously proposed. Additionally, since the problem of a lack of psychophysical laws is least threatening and the causal exclusion problem is most threatening, the exclusion problem receives the most attention. Moreover, I considered whether the problem could be overcome by justifiably denying the causal closure principle, a move that warrants the considerable attention it receives. 

The aim of this article is not to clarify which commitments IIT theorists should make, which is something IIT theorists themselves must evaluate. Rather, it provides a map of the mental causation landscape in the philosophy of the mind. Others have done substantial work on IIT and causation [10,11,12]. My contribution is to explicitly focus on the philosophical issues concerning mental causation, and to address the topic from the perspective of contemporary philosophy of mind (cf. [13]). The aim is to help clarify which philosophical commitments IIT theorists might make, and which mental causation problems will correspondingly arise, and how they might or might not be overcome. While IIT is often discussed in conversations regarding nonhuman consciousness, my focus here is human consciousness. 

## 2. Ontology of Consciousness

This article concerns the ontological identity of consciousness, which must not be confused with psychological identity. In the context of metaphysical discussions, ‘identity/identical’ refer to strict numerical ontological identity. According to Leibnitz’s law of the indiscernibility of identicals, if *x* is identical to *y* then precisely what is true of *x* at a particular time is true of *y* at that time and vice versa. In other words, if *x* and *y* are identical then *x* and *y* have all and only the same properties. This is what I mean by ‘identity’ in this article. Thus, if consciousness is identical to Ψ then what is true of consciousness is true of Ψ and vice versa. 

Some IIT adherents might be troubled by the claim that the ontological identity of consciousness needs further clarification, insisting it is already clear. After all, Oizumi et al. explicitly state:

The maximally irreducible conceptual structure (MICS) generated by a complex of elements is identical to its experience.

[14], p. 3

This suggests consciousness is identical to the conceptual structure. However, other statements by IIT theorists suggest that consciousness is a phenomenal capacity of the conceptual structure. For example, Tononi et al. write:

…we can think of the integrated information Φ^MAX^ as a measure of the intrinsic *phenomenal capacity of the conceptual structures* specified by the PSC.

[15], p. 457

Here the phenomenal capacity of the conceptual structure, which I italicized, would seem to be consciousness. However, the capacity of something and the thing that has the capacity are not necessarily identical (e.g., the desk has the capacity to support the computer, although the desk is not identical to this capacity). The authors go on to state in the immediate context: “In IIT, the experience of seeing the Sperling display is identical to a particular conceptual structure…” The careful reader will naturally wonder whether consciousness is to be understood as identical to the conceptual structure or identical to its phenomenal capacity? 

Further complicating matters, the maximally irreducible conceptual structure consciousness is often said to be identical to is elsewhere referred to as a cause-effect structure, which has cause-effect power (see [11], pp. 2–3; [2], p. 87). So IIT might be understood as claiming that there is an irreducible “conceptual” structure, which has a mathematical and abstract ring to it, and also a “cause-effect” structure, which sounds concrete and physical (cf. [2], p. 88). However, IIT proponents have simply altered how they discuss the MICS, but this demonstrates that these issues are still in development. Additionally, a further evidence of this is the different senses of ‘identity’ used to discuss IIT’s central identity claim. For example, Koch speaks of a metaphysical ontological identity, whereas Grasso clarifies that the central identity claim is not metaphysical, but explanatory (see [2], p. 88; [3], p. 52). 

Clearly, this metaphysical murkiness needs conceptual clarity. However, such a need is only to be expected, since it is natural for scientific theories generally to require refinement as time goes on. This is especially true for a theory concerning the nature of consciousness. Aristotle begins *De Anima* by pointing out that gaining any knowledge about the soul is one of the most difficult things in the world [16], p. 641. It must likewise be admitted that conceptual clarity regarding the nature of consciousness is an inevitable challenge for any view of consciousness. 

Some might object to the alleged need for such clarity by pointing out that scientific investigation often lacks conceptual clarity and yet produces pragmatic results. To borrow an example from Robert Prentner, the scientist working on a theory of combustion engines does not need much conceptual clarity about the metaphysical nature of causation to make progress, resulting in an engine that functions with more power. While it is true that conceptual clarity is not always needed to get such pragmatic results, it is needed to better understand the actual nature of an entity. If we only want to understand how to affect consciousness, conceptual clarity is not as important. However, if we want to better understand the actual nature of consciousness and its relationship to its physical substrate, conceptual clarity is needed. The more accurate our concepts about consciousness are, the more accurate our knowledge about the nature of consciousness will be. 

The following section outlines three different possible versions of IIT that make distinct metaphysical commitments concerning consciousness and causation. In due course, we’ll see that the various commitments lead to different problems pertaining to mental causation, and different possible solutions for dealing with those problems. 

## 3. Reductive, Non-Reductive, and Non-Physicalist IIT

Also at the beginning of *De Anima*, Aristotle correctly points out that one of the difficulties besetting inquiry in what we would call the philosophy of mind and science of consciousness is figuring out the appropriate method of inquiry and epistemic starting points [16], p. 641. A common contemporary epistemological assumption is that inquiry about consciousness should begin with empirical information about the brain. However, IIT’s Cartesian-Augustinian approach challenges this assumption by beginning with consciousness as a fundamental starting point (see [2], p. 2). IIT is based on five self-evident axioms about the nature of consciousness, from which corresponding postulates are inferred about the PSC [1,5,15,17]. In light of the axioms, it is postulated that the PSC will exemplify a maximally irreducible causal structure (MICS), manifesting causal power intrinsically upon itself. This structure is predicted to be exemplified by neuronal coalitions in a posterior hot zone consisting of areas in the parietal, occipital and temporal lobes of the cerebral cortex [18,19]. These neuronal coalitions are the neural correlates of consciousness, which IIT calls the physical substrate of consciousness (for brevity, PSC). 

This section outlines what I call: reductive IIT, non-reductive IIT, and non-physicalist IIT. Each is distinguished by different commitments regarding the identity of consciousness vis-à-vis the MICS and the nature of causation. These options do not exhaust the list of possibilities, and nuanced varieties of each are possible. Yet, for our purpose, outlining the basic essentials along with the potential costs and benefits that motivate each one will be most effective. 

### 3.1. Reductive IIT

No version of IIT is reductive in two respects. First, no version of IIT says consciousness is identical to its physical substrate. This makes it consistent with multiple realizability according to which systems other than our nervous systems could be the PSC. Second, the causal structure is maximally irreducible, in that it is not reducible to its constituent elements. Yet, at times IIT proponents seem to ontologically identify consciousness with the MICS, or the set of causal relations between the mechanisms constituting the MICS (see, e.g., [2], pp. 88, 164; [14], p. 3). Let’s call this view reductive IIT since it reduces conscious to the MICS. 

One motivation for adopting reductive IIT is its apparent parsimonious ontology, which seems to promise that one can remain within the philosophical orthodoxy of materialism and physicalism. This apparent benefit could be challenged (cf. [20]). However, it will be helpful to grant it here and provide a brief sketch of more pertinent problems. From the standpoint of IIT, which is committed to the real ontological existence of consciousness and the Eleatic principle, reductive IIT is risky, for reasons similar to those that make reductive physicalism precarious (see [7], pp. 118–119).

First, it is difficult to see how phenomenal consciousness can be reduced to a set of causal relations between physical mechanisms, raising the question of why these relations feel like anything, which appears like another version of the hard problem (cf. [21], p. xii; [2], p. 76). Second, a conscious subject has direct epistemic access to her conscious experience, but not the causal relations in her brain, which suggests that her consciousness is not identical to the causal structure consisting of such causal relations in her brain. As Tononi points out, our conscious experience is the “only fact” one can be “immediately and absolutely sure of” [17], p. 243. We cannot, however, be immediately and absolutely sure of any causal structure in our brain. Therefore, it appears that the conscious experience and the causal structure in the brain cannot be ontologically identical, given Leibnitz’s law mentioned above. Third, even if consciousness is identical to a structure consisting of causal relations between physical mechanisms exercising their causal power, it is the mechanisms that cause effects. Consequently, how does consciousness, which is not identical to the physical mechanisms that are the PSC, bring any new causal power into the world? It is hard to say (see Section 5). Given that and the Eleatic principle, eliminativism is lurking, for consciousness needs to produce discernable effects in order for its existence to be known (see Section 1; [2], p. 81; [4], fn 7; [5], p. 631). Additionally, it would be simpler to just eliminate consciousness, as it would add no new explanatory power. 

Thus, reductive IIT has its risks insofar as it flirts with the anti-realist views of consciousness that IIT stands squarely against, by affirming that consciousness is a real, undeniable feature in the natural world, which produces measurable effects (cf. [22,23,24] & [2], pp. 3–4). The aforementioned concerns provide motivation for non-reductive IIT.

### 3.2. Non-Reductive IIT

As previously mentioned, according to all versions of IIT, the MICS is not reducible to the physical mechanisms and the causal relations that realize it. The best rationale for why the MICS is not identical to the physical mechanisms and the causal relations between them is that there is something true of the whole causal structure that is not true of the physical parts. The MICS has causal power that the individual parts do not have [11]; [5], p. 631. It has causal power to produce effects intrinsically within the system and upon its constituent parts. Therefore, the MICS exists as an entity that is not reducible to the physical mechanisms that are its substrate (see [5], pp. 622, 631). Yet, what I will call “non-reductive IIT” not only says that the MICS is not reducible to the physical mechanisms realizing it, but also that consciousness is not identical to the MICS—at least not in the strict ontological sense clarified above (see Section 2). 

Instead, according to non-reductive IIT, there is an explanatory relation between the MICS and consciousness, so that a measurement of the former yields a measurement of the latter (cf. [3], pp. 52–53, 69). This version of IIT does not take on the burden of picking out some unlikely physical candidate to ontologically identify consciousness with. That said, there are various ways in which non-reductive IIT adherents might understand the identity of consciousness. While consciousness is not identical to the MICS, the non-reductive IIT theorist could identify it with the MICS’s intrinsic causal power, which would seem consistent with the explanatory relation mentioned above. Although this would entail that the MICS is likewise not identical to its causal power, due to the transitivity of identity (i.e., if x = y and y = z then x = z). Yet, consciousness could rather be identical to something else that is not identical to but coincides with the MICS. For example, by exploiting Heil’s view of powers, one might think that consciousness is not a power of the MICS per se, but is nevertheless a mental power that is a reciprocal partner of the MICS’s intrinsic causal power (see [25], Ch. 6). Similarly, relying on Owen’s Mind-Body Powers model of NCC, one might identify consciousness as a mental partner-power of the bodily intrinsic power manifested by the MICS (see [26,27]). This would be consistent with the fact that conscious subjects have direct epistemic access to their consciousness, but not the manifestation of biological powers manifested by neuronal mechanisms in the brain. 

These are just several ways that the ontological identity of consciousness might be understood on non-reductive IIT; they do not exhaust the options. What is essential is that consciousness is not identical to the MICS. While this position is not a reductive physicalist position, it could be categorized as a version of nonreductive physicalism insofar as it is committed to the essential tenets of physicalism, such as the causal closure of the physical domain and mind-body supervenience (see [28], pp. 209–210; [29], p. 13). Accordingly, the physical would be metaphysically fundamental and ground the mental. Such commitments qualify non-reductive IIT as a broadly physicalist position and distinguish it from non-physicalist IIT. 

### 3.3. Non-Physicalist IIT

Like non-reductive IIT, what I will call “non-physicalist IIT” also denies that consciousness is identical to the MICS. Additionally, the options mentioned in the previous section for how the ontological identity of consciousness could be understood are open to non-physicalist IIT. Where non-physicalist IIT differs from non-reductive IIT is in its commitment, or lack thereof, to physicalism’s aforementioned essential tenets—i.e., causal closure and supervenience. 

While there are various ways of understanding it, mind-body supervenience says that the physical is ontologically fundamental and the mental ontologically depends on the physical. By contrast, with respect to conscious material entities, non-physicalist IIT claims that consciousness is metaphysically fundamental vis-à-vis the physical. This fits naturally with IIT’s epistemic methodology of starting with phenomenology to formulate axioms from which empirically testable postulates about the nature of the physical substrate are inferred. Within a materialist milieu, it might be common to start the epistemic inquiry about consciousness with empirically known facts about the brain, which makes sense if the physical is metaphysically fundamental and the mental ontologically depends on it. However, IIT takes the opposite approach, which makes sense if the mental is metaphysically fundamental and its physical substrate ontologically depends on it. On non-physicalist IIT, consciousness is epistemologically fundamental because it is metaphysically fundamental and grounds the MICS realized by the PSC. In a sense, this view turns the mind-body supervenience of physicalism on its head. 

Moreover, non-physicalist IIT is not committed to the physicalist principle of the causal closure of the physical domain. This principle will be discussed at length in due course (see Section 5). For now, suffice it to say that according to the principle of causal closure there are only physical causes of physical effects, and no other causes. As we will see, this principle causes trouble for views that claim the mental is causally efficacious yet not identical to something physical. However, non-physicalist IIT avoids such trouble, given its denial of the principle. Although since the principle is widely held, rationally denying it requires sufficient warrant considered below (Section 5). Having now distinguished reductive, non-reductive, and non-physicalist IIT, let’s turn to the problems regarding mental causation, beginning with the lack of psychophysical laws.

## 4. Lack of Psychophysical Laws

The alleged lack of psychophysical laws to govern mental causation is historically connected to the anomalous monism held by Donald Davidson, who had a significant impact on 20th century philosophy of mind and action. In this section, Davidson’s anomalous monism and the way in which it leads to the problem of a lack of psychophysical laws will be presented. (For an introduction to the complexities of Davidson’s view that has informed my own articulation, see [30].) Subsequently, the threat this problem poses to the versions of IIT outlined above will be considered in Section 4.1. 

In Davidson’s view, mental events are events describable in mental terms and physical events are events describable in physical terms (see [31], p. 215). According to his anomalous monism, mental event types are distinct from physical event types, and there are strict causal laws pertaining to physical types, but no such laws regarding mental types (see [30], p. 252). Hence, Davidson thought that every token mental event that causes a particular physical event is identical to a token physical event. Yet, mental types, according to Davidson, are irreducible to physical types. So, Emma’s mental intention that causes her left arm to rise is identical to some instance of a physical event. However, the type of mental event, which Emma’s intention is an instance of, is irreducible to any physical type. In other words, the mental type ‘intention to raise left arm’ is not reducible to a specific physical type, such as ‘brain fiber-ϕ firing.’ 

Why adopt anomalous monism? At the heart of Davidson’s rationale is the idea that there are “strict laws” governing physical causation, which make causation possible between physical events, but there are no such laws governing mental to physical causation. That is, there are no psychophysical laws—i.e., laws that govern causation between mental events and physical events. This is problematic given two assumptions: *a.* the causal framework of event causation, and *b.* the nomological requirement. Those who hold to event causation view causation as a relation between events. Causation, on this framework, is an external relation requiring distinct events as relata that stand in relation to one another. The nomological assumption says: whenever one event causes another event the causal relationship is derived merely from noncausal features of the situation and pertinent covering laws. Given these assumptions, every case of causation has distinct events governed by a covering law(s). 

Now we can understand the motivation for anomalous monism. Since there are no psychophysical laws, there cannot be causation between nonphysical mental events and physical events. Thus, whenever a mental event causes a physical event, that mental event must be a physical event. Otherwise it could not cause the physical event. Reducing the specific mental event to a physical event allegedly allows one to explain why the mental event caused the physical event. Yet the idea is that mental types are still distinct from physical types. This view supposedly allows one to have mental causation without a full-blown reduction of the mental to the physical.

Given the above assumptions and Davidson’s belief that there can be no psychophysical laws, we can see why he adopted anomalous monism. However, why did he think that there cannot be psychophysical laws? Before we answer this question, let’s be clear about what a “strict law” is supposed to be. According to Davidson, laws are linguistic (see [31], p. 215). Strict laws, he thought, are true statements that are universally applicable without qualification (see [30], p. 252). Such laws allegedly allow us to explain why events take place. Given the law of gravity, for example, we can say why the coin fell to the floor when Jon released it. Whenever anyone releases a coin, while on earth, the coin will fall toward the ground. The thought is that physical laws apply universally without exception. So we can explain physical events by appeal to such laws. However, this is not so for mental events [32], p. 216. 

Davidson thought that mental concepts were “irreducibly causal” and thus cannot be specified in a way that universally applies (see [32], p. 216; [30], p. 254). This flows out of Davidson’s position known as the ‘holism of the mental.’ According to the holism of the mental, what leads someone to act in a specific way has to do with their whole psychology at that time, not particular mental events. So psychological causes of actions are not specific mental states or specific reasons. Yet, Davidson also thought psychological explanations, which are needed for strict laws, are aimed at specifying the specific reasons that cause someone to act.

Therefore a problem arises—the specific reasons that cause one to perform a given action need to be specified for there to be strict laws, but this cannot be universally specified [32], p. 216. For one, we can never identify particular reasons that cause one to act, because it is one’s entire psychology at a time that causes their action, not specific mental states. In addition, the same reasons will not always cause the same result. Basically, strict laws depend on psychological explanations that require the identification of specific reasons that universally cause specific actions, but such an identification is not possible given the holism of the mental. 

For elucidation, let’s consider an example. On the nineteenth day of April 1861, one week after confederate forces fired at Fort Sumter in Charleston Harbor, Abraham Lincoln ordered a blockade of southern seaports and the United States Civil War began. What reasons spinning around in Lincoln’s mind caused his action? Consider five hypothetical reasons: *a.* the blockade will severally damage the economy of the rebelling states; *b*. the blockade will prevent confederate forces from securing needed military supplies; *c.* instigating war with the South will lead to freedom for southern slaves; *d.* being President during a civil war will secure his place in history; *e.* not responding to the shots fired on Fort Sumter will send the message that the North is ill prepared for war. 

Given the holism of the mental, it is impossible to specify which reason (or set of reasons) caused Lincoln to act as he did. For Lincoln’s action was determined by Lincoln’s entire mental psychology at the time in question, not by any one particular reason or set of reasons. Therefore, we cannot specify which reason(s) caused Lincoln to act as he did. Additionally, even if we could, there is an additional problem. The reason(s) that caused Lincoln’s action would not cause the same effect without exception. 

Suppose Lincoln’s action was caused by reasons *a* and *c*. In such a case, for there to be a psychological law, we need to be able to say that *a* and *c* will always cause the same action of someone in Lincoln’s position. Yet, if Lincoln had one additional belief—for example, *f*. a blockade of southern ports will also damage the economy of potential allies—this may have altered his decision entirely. Additionally, suppose the First Lady, Mary Lincoln, was President and Abe was the First Gentleman. We cannot say that she would have done the same, and we especially cannot say whether reasons *a* and *c* would have caused her to do so. Psychological explanations are not like physical explanations. We cannot specify which reasons will universally, and without exception, lead to certain actions. 

Therefore, according to Davidson’s line of reasoning, there are no strict psychological laws [32], p. 216. For such laws would require us to be able to say which reasons will cause specific actions universally without exception. We cannot do so. Thus, according to Davidson’s line of thought, there are no psychophysical laws that causally relate mental events to physical events. Given that there are no psychophysical laws, there are no laws to relate mental causes to physical effects. Additionally, such laws are supposedly necessary for one event to cause another event. Therefore, there cannot be causation between mental events and physical events. That is the problem of a lack of psychophysical laws, which Davidson navigated around by identifying every token mental event that causes a physical event with a token physical event. In this way, he reduced mental causes to their physical substrate.

### 4.1. Evaluating Davidson’s Causal Commitments

If Davidson’s monist solution is the only way to avoid the problem of a lack of psychophysical laws, then it threatens all three versions of IIT outlined in the previous section. After all, no version of IIT reduces consciousness to its physical substrate, as Davidson did in reducing token mental events to token physical events. 

It is tempting to think that this problem is unproblematic for reductive IIT because it reduces consciousness to the MICS. Although it is important to keep in mind the sense in which no version of IIT (including reductive IIT) is reductive. The MICS is maximally irreducible in that it has its own existence and is not identical to the physical mechanisms which are its substrate, because it has causal power that the substrate does not have (see [5], pp. 622, 631). Given the irreducibility of the maximally irreducible causal structure, which consciousness is identical to on reductive IIT, Davidson’s route of overcoming the problem is unavailable, since he reduced the token mental events involved in causation to the token physical events that actually do the causal work. Consequently, it is the physical events that have the causal potency, which is inconsistent with IIT’s commitment to the real ontological existence of consciousness and the Eleatic principle. For, if the mental event is reduced to the physical event which causes the effect, it is the physical event that has the causal power and thus can be known to exist, in light of the Eleatic principle. Consequently, the eliminativism that IIT opposes is knocking on the door. 

Therefore, IIT proponents ought to consider alternative ways to deal with this problem. As Kim once pointed out, philosophical problems do not arise in a vacuum; they arise due to our conflicting philosophical commitments [7], p. 29. Davidson held the following commitments, which prompt the problem of a lack of psychophysical laws:

- Framework of event causation: causation is a relation between events.
- Causation is an external relation between causal relata (i.e., events).
- Causation requires strict universal laws to explain cause and effect.
- Laws are linguistic and depend on our identification of universal regularities.
- Reasons one has for a particular belief or action cause one’s belief or action.

Minus any one of these commitments, the problem fails to arise. Therefore, IIT theorists can avoid the problem via a justified denial of any one of them, as long as doing so is consistent with IIT. While I cannot here comprehensively explicate how IIT adherents might justifiably make such denials, several suggestions are worth mentioning.

To begin with, it is fair to question why laws, in Davidson’s sense, are needed for causation and how they are supposed to explain causation. According to Davidson, strict laws are essentially statements with universal applicability. Thus, laws depend on our ability to ascertain when, why, and how certain events inevitably follow corresponding events as effects. Additionally, laws are basically our linguistic statements that fittingly apply. If that is what laws are, the lack of them is inconsequential. It could be that whatever makes possible causation between a mental state and a bodily state is unascertainable. We may never know why, or how, pin pricks cause pain events. Likewise, we may never know why, or how, my intention to raise my arm results in my arm raising. Nevertheless, not knowing the answers to the “why question” or the “how question” would not threaten the fact that there seems to be something that makes possible the consistency of pin pricks causing pain events and intentions causing bodily movements. Facts about the world can be entirely unknown to us or inexpressible for us. Our inability to specify why, how, or when certain mental states will lead to physical states does not do anything to undermine the fact that they do. So a lack of psychophysical laws in Davidson’s sense of ‘law’ seems harmless to IIT.

Furthermore, it is worth questioning the framework of event causation that Davidson assumes, according to which causation depends on events externally linked by laws. The oddity of event causation is that causation is explained by things that are not themselves causal by nature. Events are not themselves causal on this view, as they need laws. Additionally, laws are likewise not themselves causal, as they need events. However, somehow causation supposedly emerges from the combination of events connected by laws. Explaining how this happens is a serious difficulty for event causation, which is the metaphysical view of causation presupposed in the rationale for the problem of a lack of psychophysical laws. 

However, non-reductive IIT and non-physicalist IIT are consistent with denying event causation, and rather, endorsing agent causation. On the framework of agent causation, agents have causal powers that produce particular effects when manifested [33,34,35]. While a chain of events can be involved in the manifestation of causal powers, it is ultimately the manifestation of such causal powers that fundamentally explains why certain effects came about. Reductive IIT might fit best with event causation, but non-reductive IIT and non-physicalist IIT are most congenial to this alternative agent causal conception of causation, since these versions of IIT are committed to the idea that there is a mental power that is not reducible to the physical substrate nor the MICS. In other words, there is a causal power that is itself causal by nature and is not reduced to that which is not causal by nature. This relates to common ground IIT shares with an Aristotelian ontology of powers [36]. If consciousness is a causal power, as understood in Aristotelian metaphysics, Davidson’s psychophysical laws are unnecessary. Aristotelian causal powers provide a basis not only for mental causation, but also general causal regularities throughout the natural world that are the focus of scientific inquiry [37]. IIT theorists who wish to draw on the metaphysics of causal powers and Aristotelianism can benefit from a research revival in this area (see e.g., [38,39,40].

Overall, the problem of a lack of psychophysical laws is threatening for IIT theorists only to the degree that they agree with Davidson’s philosophical commitments concerning causation. Reductive IIT is most susceptible to the problem, assuming that it fits best with the event causation Davidson presupposed. However, the proponent of reductive IIT who agrees with event causation can still challenge the necessity of psychophysical laws according to Davidson’s understanding of what a law is. Additionally, non-reductive IIT and non-physicalist IIT are both consistent with a denial of any one of Davidson’s philosophical commitments regarding causation, and all of them together. Given that a denial of any one of Davidson’s commitments listed above dissolves the problem of a lack of psychophysical laws, and each version of IIT can reasonably deny at least one of them, the problem does not present much of a threat to IIT. For this problem to be a piercing problem, its proponents would need to give IIT theorists compelling reasons for why they should accept Davidson’s causal commitments that cause the problem. Apart from such reasons, the problem of a lack of psychophysical laws is not too threatening. A greater threat to IIT is posed by the causal exclusion problem. 

## 5. Causal Exclusion Problem

The causal exclusion problem boils down to the mental being excluded from playing a genuine causal role. Given that every physical event has a sufficient physical cause, it is thought that mental causes are excluded from causing physical events. Metaphorically speaking, all causal jobs are filled by physical causes, so mental causes are out of work. Technically speaking, if physical event *p* is sufficiently caused by mental event *M*, the causal closure of the physical domain will be violated; yet if *p* has a sufficient physical cause, say P*, then P* would preempt *M* as the cause of *p*. In effect, P* excludes *M* as the cause of *p* (see [7], p. 37). This section explicates the causal exclusion problem, why it arises, how it threatens IIT, and how IIT adherents can respond to it. 

As things will soon become complex, a simple hypothetical example is a fair place to start. Imagine that Timothy’s mother is teaching him to give charitably. Before church, where they are invited to give charitably, Timothy’s mother hooks him up to an electrical system that shocks him. The electrical shock causes his hand to open and release money held in it. When the offering plate passes by Timothy, his mother hits a button that sends electrical currents through his body causing his hand to open, and out falls the money into the plate. Given the way that things are set up, the money falls into the plate due entirely to the physical causes. 

Timothy’s mother needs parenting classes, but she might also benefit from a class on philosophy of mind in which she can reflect on the exclusion problem. After all, she is trying to teach young Timothy to be charitable, but the causal system she set up leaves no room for genuine charity to play a causal role. The physical causes—the electrical current and the automatic neural and muscular reactions—sufficiently explain why the money falls into the offering plate. There is no room for a charitable attitude, desire, or intention of Timothy’s to play any meaningful causal role in bringing about the effect of the money falling into the plate. For, even if Timothy desires to give to the church that feeds those in need, his desire and any intention of his is not causally needed for his hand to open and drop the money into the plate. When Timothy’s mother hits the button, the money will fall into the plate wholly apart from Timothy’s charitable desire and intention. Consequently, Timothy’s mental desire and intention are excluded from having a causal impact that makes a real difference. 

The same problem arises for mental causation in general due to four principles, slightly modified from Sturgeon’s article ‘Physicalism and Overdetermination’ (see [41], pp. 413–414).

(COP) Completeness-of-Physics: All physical effects have a fully revealing, purely physical history.
(IMP) Impact-of-the-mental: Mental events cause physical effects.
(NOD) No-Overdetermination: Physical effects of mental events are not generally overdetermined.
(DISTINCT) Distinct: Mental events are not identical to physical events.

COP is also known as the causal closure of the physical, which is a principle essential to physicalism according to Kim [28], pp. 209–210. Additionally, he thought that this principle supported mind-body supervenience, which says the mental is determined by its physical base and is also essential to physicalism (see [7], p. 40; [29], pp. 8–13). While some have thought that supervenience can save mental causation on physicalism, Kim showed that it actually makes the causal exclusion problem worse, and gave an argument for the problem based on supervenience (see [7], Ch. 2; [29], p. 217). Given that the problem arises in light of principles that are essential to physicalism, Kim thought that one of the most notable developments in the contemporary philosophy of the mind was that the causal exclusion problem strikes at the very heart of mainstream physicalism (see [7], pp. 30, 39). 

IMP says that the mental causes physical effects. It is commonsensical that my mental belief ‘a boulder is about to fall on me’ and my mental desire to avoid being crushed play a causal role in bringing about physical effects, such as my legs moving in a running motion. According to NOD, physical effects that are mentally caused are not caused by more than what is sufficient to bring about the effect. DISTINCT expresses the idea that the mental is not identical to the physical, which is consistent with nonreductive physicalism that says the mental is irreducible to the physical, property dualism that claims mental properties are sui generis properties, and various versions of substance dualism that claim there are nonphysical mental substances. 

It is commonly thought that any set of three of the principles is consistent, but the set of four is inconsistent (see [41], p. 414). If it were true that every physical effect had a fully revealing purely physical causal history, then no mental cause could play a causal role in bringing about any physical effect, as long as mental events are distinct from physical events. One might think physical events could just have multiple sufficient causes. Like a ship that sinks due to multiple holes that could sink the ship on their own. However, if this were so, there would be overdetermination, and NOD would be false. It seems that the set of all four principles is simply inconsistent, which motivates a denial of at least one principle. 

Despite the fact that the mental seems causally efficacious based on everyday experience, the epiphenomenalist denies IMP, surrendering the mental to the category of the causally impotent. This route is unavailable to IIT adherents, since it would entail the nonexistence of consciousness in light of the Eleatic principle and consequently undermine the first axiom of IIT. Additionally, if the first axiom is false due to the nonexistence of consciousness, the science of consciousness would be a scientific study of something that does not exist. 

The reductive physicalist denies DISTINCT and claims that the mental is identical to the physical. There are various ways this identity relation might be made—for example, one might say individual token mental states/events are identical to individual token physical states/events, or one might identify mental types with physical types. Regardless, this route too seems unpromising for all versions of IIT. For one, all version of IIT deny that consciousness is reducible to its physical substrate. Even reductive IIT denies this (recall that the MICS consciousness is said to be identical to is the maximally irreducible causal structure). Furthermore, if consciousness were identical to the physical, which is what is producing the causal effects, wouldn’t it be simpler to eliminate the mental as the Churchland’s recommend and IIT proponents oppose (see [22,23] & [2], pp. 3–4)? After all, the physical that the mental is said to be identical to would be what is causing all the effects, so it is hard to see how consciousness would be making a causal contribution that makes its existence evident and worth postulating. This would make one wonder what the science of consciousness is studying that physics is not. To boot, such a reduction seems inconsistent with IIT’s acknowledgement that consciousness is directly accessible to the conscious subject, whereas the physical substrate is not (see Section 2). Moreover, the intrinsicality problem that Mørch points out might also rule out a denial of DISTINCT as a viable option for IIT [42].

A third option is to deny NOD and posit that physical effects are often overdetermined by sufficient mental and physical causes. However, denying this principle is once again risky for IIT, for the existence of consciousness is predicated upon the fact that it makes a discernable causal contribution in the physical world. Yet, if there is a sufficient physical cause of effect as well as a sufficient mental cause of effect, how exactly are we to know that the mental cause is a co-cause of effect? If IIT avoids the exclusion problem by denying NOD, then IIT will hinge upon the strength of the answer to this question. Furthermore, it seems that if consciousness is not identical to anything physical and thus DISTINCT is true, then denying NOD would also entail a denial of COP. (However, as clarified in Section 5.2, some articulations of closure are thought to permit overdetermination involving mental causes.) For, given DISTINCT, the mental is not identical to the physical, but if NOD is false while IMP is true, then the mental must cause physical effects, even though it is not the only cause. Hence, it seems that denying NOD also entails a denial of COP. However, a denial of COP alone would suffice for dissolving the causal exclusion problem. Additionally, if IIT justifiably denied COP alone, its vitality would not hinge on an answer to the above question.

Therefore, it is worth exploring warrant IIT proponents can appeal to for denying COP and admitting causal openness between the mental and the physical, even though the mental is not identical to the physical. 

### 5.1. Reconsidering Causal Closure

Physicalists might think the physicalist framework provides a natural starting point for considering the nature of causation generally, and specifically the causal closure principle. However, as Kim points out, a different view seems to be the default, natural position: “We commonly think we, as persons, have both a mental and a bodily dimension—or, if you prefer, mental aspects and material aspects. Something like this dualism of personhood, I believe, is common lore shared across most cultures and religious traditions…” [43], p. 65 (see also [44], pp. 21–22). Moreover, it seems to us that the mental aspect that is distinct from the physical brings about physical effects as we act as agents. This is why epiphenomenalism “strikes most of us as obviously wrong, if not incoherent; the idea that our thoughts, wants, and intentions might lack causal efficacy of any kind is deeply troubling, going against everything we believe about ourselves as agents and cognizers” [6], p. 70. Although it could be false, the view that human persons are physical beings that nevertheless have a distinct mental aspect that is causally responsible for physical effects, seems to be a commonsense position that is widely held. Hence, I will call it the commonsense view. 

Chisholm noted at the outset of *Person and Object: A Metaphysical Study* that what we are justified in assuming when not philosophizing, we are justified in assuming when doing philosophy [45], p. 16. In other words, that which we are justified in assuming when not theorizing, we are justified in assuming when doing theoretical work. Perhaps there is little awareness of it, but this is common practice, not only in philosophy, but also science. For example, when the scientist crosses the street from her bus stop to get to her lab, she assumes that her sense perceptions allow her to reliably (albeit not infallibly) observe the world around her. Hence, if she sees oncoming traffic, she does not cross the road; if she sees no oncoming traffic, she crosses. Likewise, as she observes physical phenomena in her lab or while collecting data out in the field, she does not first formulate an argument for why she can trust her sense perception. Rather, just as when she was crossing the street, she assumes that her sense perception allows her to observe the world as it is. Granted, she might need tools to help her do so, and her provisional conclusions might need later revisions, but it is a fair place to start when doing empirical research. 

Of course, our philosophical presuppositions informed by the way things appear to be are not infallible. They can be proven false. However, where else shall we start but with how the world, and in this case ourselves, appear to be? Later investigations can then tell us if there are good reasons to revise or abandon our starting presuppositions. 

The commonsense view of ourselves implies that the mental, although distinct from the physical, nevertheless produces physical effects, which suggests that causal closure is false. Clearly, the causal exclusion problem calls this commonsense view into questions, but it does so by leaning on the causal closure principle as a key premise, and it is fair to ask why this premise is justified. This is especially true given that the commonsense view, which suggests closure is false, provides a reasonable place to start. If we have good reasons to accept the premises prompting the causal exclusion problem, then we have good reason to deny the commonsense view. However, if we do not have good reasons to accept the premises, then the argument does not provide rationale for denying the commonsense view, which challenges causal closure.

This is important because the opposite is often taken for granted in a materialist milieu in which the doctrines of physicalism are assumed orthodoxy (see [46], p. 1; [47], p. 152; [48], p. xii). Additionally, as Bonjour suggests, our starting points can influence what we think about the truth or falsity of causal closure:

It seems utterly obvious that mental states do causally affect the material realm…*If* a materialist account of conscious states is correct, then the principle of causal closure seems likely to be true. But if no such account is correct, then the principle is almost certainly false.

[49], p. 6

Philosophers, neuroscientists, and physicists might assume a reductive materialist ontology that implies causal closure. However, given that serious arguments have been given by leading contemporary philosophers for nonphysicalist and dualist views of the mental, it is not at all clear that one is warranted in simply assuming that materialism or causal closure are true (see [21,33,50,51,52,53,54,55]). One needs independent reasons apart from an assumption of materialism for accepting causal closure. 

Therefore, let’s now analyze the viability of independent rationale for causal closure. It is often thought that causal closure is a clear principle supported by empirical science. To the contrary, I will argue that: (1) the principle is ambiguous, which makes it difficult to analyze the justification offered for it, and (2) empirical science does not, and cannot, confirm it.

### 5.2. Causal Closure’s Ambiguity

A prerequisite for justifying a claim is specifying the claim to be justified. If you do not know what the claim is, how can you know whether the rationale offered for it actually supports it? Thus, the first step in justifying the causal closure principle is clarifying what exactly it claims. As will become apparent, this is no easy task. Satisfying this prerequisite for justifying causal closure is made difficult by the variety of ways the principle is formulated and the different ways that a key term can be understood. 

After surveying five different versions of the principle, Lowe aptly remarks: “One might have hoped for more exactitude and agreement amongst physicalists when it comes to the formulation of a principle so central to their position” [56] p. 574. For a small sampling, consider the following versions.

*Every physical effect has a sufficient physical cause*. Papineau has presented this version, which is compatible with overdetermination.

[57], p. 375 (cf. [58], p. 99)

*The chances of physical effects are always fixed by sufficient physical causes*. In an endnote following the above version, Papineau assumes that his reader asks about quantum indeterminacy and he indicates that closure can be put this way.

[57], p. 386. More so than the above version, this version suggests determinism

*At every time at which a physical event has a cause it has a sufficient physical cause*. Gibb points out this articulation of closure.

[59], pp. 2, 12. This version also permits overdetermination

*Every physical effect has its chance fully determined by physical events alone*. Noordhof prefers this version, which does not permit overdetermination and suggests determinism.

[60], p. 367

The variety of versions confuses matters, but perhaps we could just pick one version that is agreed on by most physicalists to satisfy the prerequisite of specifying the principle to be justified. Kim thinks most ontological physicalists will accept the following:

(COP*) If a physical event has a cause at *t*, it has a sufficient physical cause at *t*.

[61], p. 38

This principle seems straightforward enough, until we ask: What is meant by ‘physical’ [41]? The answer is anything but straightforward. (Sturgeon is particularly insightful here and has informed my foregoing considerations, see [41]).

Suppose that at a particular time *t_0_* you have two physical items. The first is a log with a circumference of one meter and a height of one meter. The second is a flat square board with a length of one meter and a width of one meter. While it is clear that you have two physical objects at *t*_0_, suppose that at a later time *t*_1_ you place the log under the center of the board. So, at *t*_1_ the board rests on the log and forms a table, T. Do you now have three physical items—the log, the board, and a table? Or do you just have one physical item, the table? Or do you still just have the two original physical items, the log and the board? Various philosophers, even various physicalists, will give various answers. The same would be true if our example pertained to fundamental physical particles, rather than a log and a board. Such puzzles may seem irrelevant, yet there are genuine implications for the causal closure principle.

In the causal closure principle, the term ‘physical’ can mean *microphysical* or *macrophysical* [41]. If microphysical is meant, the claim is that every microphysical event has a sufficient microphysical cause. Additionally, what would count as ‘physical’ would be fundamental physical particles (whatever they happen to be). If macrophysical is meant, the claim is that macrophysical events have sufficient macrophysical causes. Additionally, entities composed of more basic fundamental physical particles could be concrete particulars that could causally produce effects. Simply put, if ‘physical’ means microphysical then our table, T, does not count as a physical entity that exists; but if ‘physical’ means macrophysical, then it does.

Therefore, if what counts as physical, according to COP*, is microphysical fundamental physical particles, this appears to be inconsistent with the existence of macrophysical things, such as planets, animal bodies, biological organs, plants, ecosystems, tornadoes, automobiles, jet airplanes, and so forth. These seem to be physical things, although they are not microphysical things. So if the only things that count as physical entities are microphysical entities, according to the principle, then the principle seems to be at odds with the apparent existence of non-microphysical, macrophysical entities.

Furthermore, it is not just that macrophysical things seem to exist, but they also appear to causally produce physical effects that the principle does not properly acknowledge, if ‘physical’ only means microphysical. For even the most hardcore reductivist looks both ways for automobiles not subatomic particles before crossing the street. After all, it is the former that can cause bodily harm. Consider also jet engines that propel an airplane from one location to the next, a heart that pumps blood, a river current that causes erosion, the movement of a runner’s legs propelling her forward, the ozone layer absorbing solar radiation, and the like. It seems that these are physical entities, but not microphysical entities, which cause physical effects in the world. Thus, if ‘physical’ in COP* only means microphysical, then the principle does not properly address macrophysical causes of physical effects, which appear to exist.

Moreover, it is also reasonable to think that there are macrophysical causes of microphysical effects, which would be inconsistent with COP* if ‘physical’ means microphysical. While there are many examples one might give, I’ll give just two brief examples here of apparent macrophysical causes of microphysical effects that present prima facie difficulties.

First, consider a fundamental physical particle, P, which is sitting on the surface of my floor (let’s call its location *L*^1^) at time *t*_1_. Suppose that I move a vacuum cleaner hose over *L*^1^ and the vacuum inside the hose lifts P upward into the hose causing it to change locations to *L*^2^ inside the hose, where it is located at time *t*_2_. It seems that the cause of P moving from *L*^1^ to *L*^2^ is the vacuum inside the vacuum hose. Additionally, it is difficult to reduce a vacuum to a fundamental physical entity, because a vacuum requires a whole system of physical parts to be co-operative. The vacuum might be reducible to a structured collection of fundamental physical entities functioning in tandem. However, such a collection does not seem to be a fundamental microphysical entity, and therefore if it is the cause of P moving from *L*^1^ to *L*^2^ it provides a counterexample to COP* if ‘physical’ means microphysical.

Second, the natural function of a human brain requires countless individual oxygen molecules consisting of two oxygen atoms, O^2^. For simplicity’s sake, let’s assume an atom is a fundamental physical entity, and thus microphysical. Through repetitive systolic contractions, the human heart pumps blood carrying erythrocytes including hemoglobin with oxygen molecules consisting of oxygen atoms. We can imagine an individual oxygen atom, which is a constituent of an oxygen molecule, that is in a heart chamber at one time and then subsequently moves to the brain because the heart pumps the blood carrying the oxygen molecule, including the individual atom to the brain. Without the heart (or an artificial heart) pumping the blood, the movement of such atoms to the brain would cease, and the natural function of the brain would cease. The causal explanation of the movement of the individual oxygen atom seems to include a macrophysical cause—the heart’s contractions. Consequently, it appears to be an example of a macrophysical cause of a microphysical effect, which COP* understood in terms of ‘microphysical’ would not permit.

One might respond and say that the heart pumping blood is nothing more than the combination of more fundamental physical things, such as heart chambers, composed of heart tissue, composed of cells, and so on, down to the most fundamental physical particles, that are structured in a particular way. This is a reasonable response. However, even if this is true it is still the *structured combination* of these things that performs the physiological process that causes blood to be pumped and the oxygen atom considered to be transported. Such a structured combination is not a microphysical entity.

To recap, if ‘physical’ means microphysical, COP* has several prima facie difficulties. In addition to microphysical entities, our world appears to include macrophysical entities, and there appear to be macrophysical causes of macrophysical effects, as well as macrophysical causes of microphysical effects. Collectively, these issues suggest that it may not be wise to articulate COP* in terms of microphysical.

Therefore, the physicalist may want to define ‘physical’ in terms of macrophysical. Then, COP* would say all macrophysical events have sufficient macrophysical causes. However, as Sturgeon points out, if ‘physical’ means macrophysical, then the closure principle isn’t supported by commonsense nor any scientific theory [41], p. 416. Rather, “everyday experience indicates that mental events have macrophysical effects. So does macro science” [41], p. 416. Regarding the first point that experience suggests the mental produces physical effects, we must remember that DISTINCT is prima facie plausible (see Section 5.1). As Sturgeon remarks: “Mental and physical events are distinct. This is how reality strikes us pre-theoretically” [41], p. 414. The exclusion argument might disprove DISTINCT. Yet it cannot be presupposed at the outset that DISTINCT is false. To the contrary, it seems true, and given IMP it appears plausible that mental events cause physical events (see Section 5.1).

Moreover, as Sturgeon points out: “No working scientific theory says broadly physical [i.e., macrophysical] effects have fully revealing broadly physical histories” [41], p. 416. In other words, science does not say macrophysical events like handshakes, or the act of signing a contract, have merely physical causes. In fact, a mental awareness of what one is agreeing to and their mental intention to agree to it by physically signing a contract is arguably constitutive of the act itself. If I mentally intend to sign my autograph for a philosophy fan (which is quite unlikely), then it is a different act than signing a contract, even though the physical motion of my signing is the same. Additionally, macro science does not rule my mental intention out of playing a causal role. So, if ‘physical’ means macrophysical, it is difficult to see how closure is supported by either everyday experience or science.

Demonstrable justification of the causal closure principle requires clarity regarding what the principle actually claims. Such clarity is wanting and difficult to secure. Not only are there various versions of the principle with different entailments, the principle’s overall meaning vitally depends on the meaning of ‘physical.’ Yet, serious problems arise when it comes to whether ‘physical’ means microphysical or macrophysical. Only the term ‘physical’ has been considered here, but another key term—i.e., ‘cause’—would no less require clarity and would present just as many difficulties, if not more. If one is to accept the principle of causal closure, they’re due at least a clarification of what is meant by the principle, which is a tall order.

### 5.3. Empirical Support?

While clarifying the meaning of the causal closure principle is difficult, and problems arise whether ‘physical’ means macrophysical or microphysical, some might insist that there’s nevertheless strong empirical justification for the principle, despite its ambiguity. If so, that would help explain why many contemporary philosophers and scientists give credence to the principle. So, for the sake of argument, let’s assume the prerequisite of clarifying the principle could be met, and consider the merit of justifying closure on the basis of empirical investigation.

In *Philosophy of Mind*, Kim describes the causal closure of the physical domain in the following way:

Pick any physical event—say, the decay of a uranium atom or the collision of two stars in distant space—and trace its causal ancestry or posterity as far as you would like; the principle of physical causal closure says that this will never take you outside the physical domain. Thus, no causal chain involving a physical event ever crosses the boundary of the physical into the nonphysical: If *x* is a physical event and *y* is a cause or effect of *x*, then *y* too must be a physical event.

[29], p. 214

Kim isn’t intending to offer an argument for closure. Yet, his words echo a common argument, according to which our empirical observations only reveal physical causes of physical phenomena, and therefore causal closure is true. In other words, if closure is true, the empirical investigations of physical scientists will only reveal physical causes, which is the case, and therefore closure is true.

There are multiple reasons this is poor justification for causal closure. To begin with, modern scientists do not often see it as their job to identify anything but physical causes of physical phenomena, so they do not focus on searching for any other causes such as mental causes. This is partly why consciousness studies were considered taboo during the 20th century, and the contemporary science of consciousness struggled to get off the ground (cf. [62], p. 1). Given that the empirical investigations of physical scientists are aimed at identifying physical causes, it is inconsequential and tautologous that their investigations lead only to physical causes. That is, after all, what their investigations are aimed at identifying. Only if one presupposes that the physical sciences give an exhaustive account of the world—and therefore begs the question by indirectly assuming causal closure—does it make sense to conclude that there are only physical causes because physical scientists identify only physical causes.

Secondly, our understanding of the most likely locus of mental causal effects—i.e., the human brain—is far from exhaustive. While critiquing computationalism, Koch highlights the limited extent of our current knowledge:

The dirty secret of computational neuroscience is that we still do not have a complete dynamic model of the nervous system of the worm *C. elegans*, though it only has 302 nerve cells and its wiring diagram, its connectome, is known. So here we are, trying to understand the human brain, when we do not yet understand the worm brain.

[2], p. 138

Koch is the President and Chief Scientific Officer at the Allen Institute for Brain Science, which endeavors to map not only the mouse brain but also the human brain. If anyone knows the current status of our knowledge about the brain, he does. In the above quote he is not commenting on causal closure, but his point has implications for the justification offered for closure. If there are mental causes of physical effects that are irreducible to physical events, they are most likely in the human brain, which is still full of mystery at this point. Although our knowledge is steadily growing, it is significantly premature to claim that all events in the brain have a fully revealing physical causal history.

Furthermore, our lack of knowledge only increases as a problem for causal closure when one considers what Ellis calls “Crick’s fallacy” [63], p. 375. Crick claimed that human persons and their mental life are nothing more than “the behavior of a vast assembly of nerve cells and their associated molecules” [64], p. 3. Commenting on Crick’s claim, Ellis asks why Crick stopped the reductive process at the level of cells, rather than going all the way down to the level of particle physics? The reason, according to Ellis, is that Crick knows that nerve cells have causal power to produce physical effects. “I agree,” writes Ellis, before continuing:

But the implication is that they have causal powers over their constituent atoms, protons, and electrons, in order to make their own causal powers effective. He is assuming the viability of top-down causation to lower levels, a proof that it exists and is significant in the operation of the brain. But if we accept this, we must recognize that assigning real causal powers to an intermediate level such as nerve cells and their associated molecules, acting top-down on the levels below in order to have this power, only makes sense if we assign real causal powers to every other level as well…And then there is no reason to deny the reality of causal powers of your joys and your sorrows (the emotional systems), or your memories (allowing the predictions that underlie our sensory systems) and ambitions (your set of goals that are the decision framework for what you do).

[63], p. 375

According to Ellis, Crick’s reductive stopping point at the cellular level is arbitrary and inconsistent. Nevertheless, even where Crick does stop and what he tacitly admits is inconsistent with causal closure in its most defensible form, in which ‘physical’ refers to microphysical. Kim thinks causal closure implies supervenience and that the higher levels are determined by fundamental physical levels (see [7], p. 40; [29], pp. 8–13). Given that, the idea that a neuron, which is at a nonfundamental physical level, could have causal power at its own cellular level, much less a lower level, stands in opposition to causal closure. Yet, if neurons do not have such causal power, why study neurons? Assuming that all the causal action is at lower levels, neuroscientists cannot be studying entities that make a real causal contribution to the world.

Another independent reason as to why the justification appealing to empirical investigations fails is demonstrated by Lowe’s rebuttal, which can be summarized as follows [65]. Suppose there is a physical event *E* that necessitates two co-causes that are always co-actualized. Suppose further that one of the necessary co-causes is physical and observable, while the other is nonphysical and invisible. Let us use ‘*O*’ to designate the observable cause and ‘*i*’ to designate the invisible cause. Accordingly, *O* and *i* inevitably cause *E*. So, when *O* and *i* occur, *E* occurs. Let us represent the occurrence of the co-causes and the subsequent effect as: (*O* and *i* ⇒ *E*). The tricky part is that when (*O* and *i* ⇒ *E*) obtains, *i* will not be recognized by empirical investigation (since it is nonphysical and invisible), but *O* will be (as it is physical and observable). Therefore, whenever (*O* and *i* ⇒ *E*) obtains it will appear to observers as though (*O* ⇒ *E*) obtains. Consequently, it will appear as though *O* is a sufficient lone cause of *E*, even though it is not. For the other necessary co-cause *i*, which happens to be nonphysical and invisible, is also always present, despite appearances. One might think we could deduce that an indivisible co-cause is also making a causal contribution if there were some cases where *O* was present along with *E*, and other cases where *O* was present and *E* was absent. However, given that the two co-causes are always co-actualized together, such a scenario would never happen. As a result, *i* would not be empirically detectable.

The basic worry is that if there were an invisible nonphysical co-cause of *E* that was making a causal contribution in bringing about *E*, it could not be known on the basis of empirical investigation; nor could it be known that the observable cause is not itself sufficient. For the observable cause would appear to be the lone sufficient cause from the vantage point of empirical investigation, whether or not it actually is. Note that even if such a scenario is not actual anywhere, but merely possible, it undermines the legitimacy of inferring that there are no nonphysical causes on the basis of such empirical investigations. Thus, such investigations cannot, in principle, prove that there are only physical causes. Put differently, empirical investigations cannot possibly support causal closure.

While the causal closure of the physical domain is a widely accepted principle and arguably an essential doctrine of physicalism, it is unclear how empirical science is supposed to support it. Hence Bonjour’s puzzlement:

Clearly this ‘principle’ is not and could not be an empirical result: no empirical investigation that is at all feasible (practically or morally) could ever establish that human bodies, the most likely locus of such external influence, are in fact never affected, even in small and subtle ways, by non-material causes. We are told that scientists accept this principle, and often that most philosophers accept it as well. But do they have any compelling reasons for such acceptance? Or is this vaunted principle nothing more than an unargued and undefended assumption—a kind of intellectual prejudice, in the literal meaning of the word?

[49], p. 6

Since physicalism is still the dominant philosophical position, some might wish to maintain a commitment to causal closure to remain within the physicalist camp (cf. [28], pp. 209–210). However, Kim has made one thing clear—“Physicalism cannot be had on the cheap”—which the exclusion problem prompted by closure makes evident [7], p. 120. However, minus causal closure, the exclusion problem dissipates. Additionally, minus a good reason why IIT theorists should saddle themselves with the principle and consequently the problem, they can rebut the argument for the exclusion problem by denying the principle. Additionally, as the foregoing considerations suggests, the justification for it is wanting. (For further challenges to the causal closure principle and its applicability to mental causation, see [55,66,67,68,69].)

Moreover, proponents of IIT have actually provided theoretical research supporting conclusions that counter causal closure [10,11,12]. However, when it comes to refuting the argument for causal exclusion, they must be clear about how it deflates the problem. Namely, it justifies denying a key premise—i.e., COP. For example, what Hoel et al. call “supersedence” entails a denial of supervenience and causal closure, which is justified, but must be explicitly acknowledged to demonstrably refute the exclusion problem (see [12], p. 19795). And Baxendale and Mindt argue that interventionism gives IIT the resources to solve the problem [13]. But their solution involves denying supervenience, which Kim considered essential to physicalism and implied by causal closure (see [7], p. 40). Additionally, if closure implies supervenience, a denial of supervenience implies a denial of closure.

Apart from a denial of causal closure, it is difficult to see how any version of IIT outlined in Section 2 can overcome the causal exclusion problem. That said, insofar as reductive IIT and non-reductive IIT remain broadly physicalist views by remaining committed to physicalism’s essential doctrines, this option is unviable. However, since the causal closure principle is not essential to IIT itself and non-physicalist IIT is not committed to it, non-physicalist IIT can evade the exclusion problem via a justified denial of the principle.

## 6. The Causal Pairing Problem

An upshot of the causal exclusion problem is that “abandoning the substantival dualism of Descartes does not get us out of the woods as far as mental causation is concerned” since the physicalist tenets of causal closure and supervenience lead to the problem [7], pp. 39, 30. Given this, some nonreductivists have considered abandoning physicalism and reconsidering dualist alternatives. Since they find the route of reductive physicalism unpalatable as it does not seem to preserve genuine sui generis mental causation—hence, Kim tries to show via the causal pairing problem that dusting off dualism will only make matters worse [6], pp. 70–71.

The causal pairing problem is Kim’s development of Princess Elisabeth of Bohemia’s famous objection to Descartes’ mind-body view [6,61,70]. Kim argues that mental causation is impossible for a nonphysical mind [6], Ch. 3. The rationale he gives rests on the premises that every cause is paired with its effect, and spatial relations are the only relations that pair causes with their effects. Given that nonphysical minds or nonphysical mental states are not spatial, they cannot stand in spatial relations, and therefore cannot be causally paired with effects. Consequently, mental causation for such minds and mental states is impossible. That is the causal pairing problem. Since reductive IIT identifies consciousness with the set of causal relations constituting the MICS, it does not face this problem. However, the causal pairing problem does arise for non-physicalist IIT, as well as non-reductive IIT, which becomes apparent in light of how exactly the pairing problem threatens mental causation in two directions.

### 6.1. Threatening Mental-to-Physical and Physical-to-Mental Causation

While the causal pairing problem is usually discussed in relation to Cartesian dualism, the problem arises for any view that says the mental is not something physical and yet stands in a causal relation. Consequently, it is a problem not only for downward mental-to-physical causation, but also for upward physical-to-mental causation. For the linchpin is: only that which is physical can stand in the spatial relations required for causal pairing. Notice that, if this is true, it would apply whether the causation in question is downward, from the mental to the physical, or upward, from the physical to the mental. So this problem arises for any nonreductivist view that says the mental is not something physical and yet it produces physical effects, as well as the nonreductive epiphenomenalist who says the physical base causes mental events or states that are not something physical. For the problem is that the mental cannot stand in any causal pairing relation due to its inability to stand in spatial relations required for causal pairing relations. Thus, the pairing problem is actually broader than often thought, since it applies to causation in both directions, from the mental to the physical and vice versa. Hence, the argument can be formally presented like so:

(CP) Causal Pairing: Every cause must stand in a cause-effect pairing relation with its effect.
(SR) Spatial Relations: Every cause-effect pairing relation requires a spatial relation.
(NOS) Nonspatial: Nonphysical minds/mental states are not spatial.
(NP) No pairing: Nonphysical minds/mental states cannot stand in cause-effect pairing relations.
(NC) No cause: Nonphysical minds/mental states cannot cause effects.
(NE) No effect: Nonphysical minds/mental states cannot be the effect of a cause.

As this formalization makes apparent, if the argument is successful, it negates mental-to-physical causation for nonphysical minds or mental states, but also physical-to-mental causation where the mental state is the effect of its physical cause. Nevertheless, whenever we have our blood drawn or get a tattoo, we are reminded that pinpricks cause pain. That is, physical events can cause mental states. This forces even the nonreductive physicalist who claims the feeling of pain is not reduced to the physical to face the causal pairing problem. For if only that which is physical can stand in spatial relations and therefore pairing relations and therefore causal relations, then the nonphysical mental state of feeling pain cannot be caused by the physical base. (Granted, the nonreductive physicalist could claim the physical-to-mental relation is a noncausal supervenience relation, making the need for causal pairing obsolete. Yet, there are two relevant points to consider. First, such a move will lead to the exclusion problem discussed above. Second, as the nonreductive physicalist might deny the first premise by appealing to supervenience, one can deny the second by an appeal to grounding, as suggested in Section 6.2 below.)

The way in which the pairing problem arises for non-reductive IIT is now apparent. According to non-reductive IIT, consciousness is not identical to something physical, and yet it causes physical effects. However, if consciousness is not reduced to something physical, how does it stand in the requisite causal pairing relations that hinge on spatial relations? Assuming that spatial relations are necessary to pair causes with effects, non-reductive IIT must answer that question to overcome the causal pairing problem. I do not see how non-reductive IIT could answer that question. However, the situation is different for non-physicalist IIT.

### 6.2. Grounding Causal Pairing

Non-physicalist IIT can challenge Kim’s claim that spatial relations are the only relations that can pair cause and effect. Elsewhere, I have argued at length that a hylomorphic understanding of human ontology can circumnavigate the causal pairing problem by appealing to a grounding relation and that IIT could follow suit [9]. While I cannot provide an adequate explication here, a brief outline is worthwhile. The central move is providing an alternative type of relation that can pair cause and effect—namely, a grounding relation that the mental and its physical substrate stand in that is metaphysically prior to any causal relation. This move is inspired by Thomas Aquinas.

Rather than following Plato, who thought the soul is united to the body as mover to moved, or cause and effect, Aquinas followed Aristotle’s thought that the soul is “immediately united to its body as form to matter” [71], 1a 76.3c, 76.7c. According to Aristotle’s hylomorphic understanding of substances, all material substances consist of unified matter, and what unites it is a form. The human soul, on Aquinas’s hylomorphic view, is united to its body as the form of that body that unifies its matter [71], 1a 76.6 ad 3. As the form of the body, the soul grounds the existence of the body as one unified human body.

On this hylomorphic view, the soul stands in a grounding relation to the body, and this grounding relation is a noncausal explanatory relation with several characteristics (see [9], sect. 2.4). To begin with, a grounding relation is asymmetric. So if Φ grounds Ψ, then Ψ does not ground Φ. Correspondingly, a grounding relation involves dependency so that if Φ grounds Ψ, then Ψ depends on Φ and not vice versa. Additionally, if Φ grounds Ψ, then Φ is explanatorily prior to Ψ. That is not to say that Φ is temporally prior to Ψ, but that Ψ is true in virtue of Φ being true, and thus Φ is more ontologically fundamental than Ψ. The soul, according to hylomorphism, is ontologically fundamental vis-à-vis the body. Additionally, the grounding relation the soul and body stand in is explanatorily prior to any soul-body causal relation. Given that the soul grounds the existence of the body on this view of human ontology, there is a very natural explanation for why a person’s soul would be causally paired with their body.

To understand the explanation, it is helpful to consider an example Kim gave to illustrate the causal pairing problem (see [6], pp. 76–77). Imagine two unfortunate fellas, Smith and Jones, who are psychologically synchronized, so that whenever Smith wills to raise his right hand, Jones does the same. As a result, whenever Smith’s right hand goes up, so does Jones’. Correspondingly, whenever Jones wills to raise his hand, Smith does likewise, and their hands simultaneously rise. This raises Kim’s key question: “So why is it not the case that Smith’s volition causes Jones’ hand to go up, and that Jones’ volition causes Smith’s hand to go up?” [6], p. 76. In other words, why is the volition of Smith’s soul causally paired with his hand raising, but not Jones’? If their souls were spatially located, then the bodily movements could be paired with their mental causes based on spatial relations. Additionally, Kim suggests that only spatial relations could serve to pair a cause with its effect.

However, the hylomorphist has an alternative to spatial relations that can explain why the mental volition of Smith’s soul is causally paired with his hand rising and Jones’ is causally paired with his hand. Smith’s soul is the form of his body and thus grounds the existence of his body, not Jones’; and Jones’ soul is the form of his body and thus grounds the existence of his body, not Smith’s. Given this, it is only fitting that Smith’s soul is causally paired with Smith’s body, not Jones’. Likewise, since Jones’ soul grounds the existence of Jones’ body, not Smith’s, it is only fitting that his soul is causally paired with his body, not Smith’s. In short, the hylomorphist can deny the second premise, SR, in the argument above (see Section 6.1) by appealing to a grounding relation (rather than a spatial relation) to explain mental causal pairing.

The proponent of non-physicalist IIT could potentially do likewise, assuming that consciousness is more ontologically fundamental than the PSC and grounds its existence. According to physicalism, physics is ontologically fundamental, but according to non-physicalist IIT, consciousness is ontologically fundamental vis-à-vis the PSC and grounds the existence of the PSC. On this framework, it is not consciousness that ontologically depends on its physical substrate for its existence but just the opposite—the PSC is grounded by consciousness. This fits the integrated information theory’s epistemic methodology of starting with axioms about consciousness and then inferring what is probable about the PSC. However, it also provides a grounding relation that can act as an alternative to spatial relations for pairing mental causes with physical bodily effects. The non-physicalist IIT theorist could explain why a mental event is causally paired with a physical event in the MICS by appealing to the more fundamental grounding relation the MICS and the consciousness grounding its existence stand in. Simply put, since the latter grounds the former, they are naturally causally paired. Given that, SR in the argument above is negated and consequently NP, along with NC and NE, fail to follow.

Of course, there is more to be said, so I again refer interested readers to [9], where I give a further explanation of hylomorphism, grounding, and how they relate to causal pairing in mental causation. In sum, the causal pairing problem is not a problem for reductive IIT, but it is a problem that non-reductive and non-physicalist IIT face. Yet, as I have suggested here and argued at length elsewhere, non-physicalist IIT can circumnavigate the causal pairing problem. Some IIT theorist might be reluctant to embrace non-physicalist IIT assuming it would compromise IIT’s promise of providing a basis for empirically discerning and quantifying consciousness. I address this concern in [27], where I argue that the Mind-Body Powers model of neural correlates of consciousness (NCC) combined with IIT’s prediction about the full NCC preserves the possibility of empirically discerning and quantifying irreducible consciousness (see also [26]).

## 7. Conclusions

In this article, I have surveyed three paramount problems regarding mental causation in contemporary philosophy of mind and how each problem might threaten the causal efficacy of consciousness on IIT. After outlining possible versions of IIT consisting of different metaphysical commitments, I explicated the three problems and the threat each poses. First, the alleged lack of psychophysical laws was considered and found to hardly threaten all three versions of IIT. The causal exclusion problem, on the other hand, presents a significant challenge to reductive IIT and non-reductive IIT. However, non-physicalist IIT can evade the problem by a warranted denial of the causal closure principle. The causal pairing problem does not pose a threat to reductive IIT, but it does threaten non-reductive and non-physicalist IIT. Nevertheless, non-physicalist IIT can account for causal pairing via a mental-to-physical grounding relation that turns the ontological priority of physicalism’s mind-body supervenience on its head. I trust that this survey can aid IIT theorists as they further develop and hone the metaphysical commitments of IIT.
